# Identification of new putative driver mutations and predictors of disease evolution in chronic lymphocytic leukemia

**DOI:** 10.1038/s41408-019-0243-3

**Published:** 2019-09-30

**Authors:** Adrián Mosquera Orgueira, Beatriz Antelo Rodríguez, José Ángel Díaz Arias, José Luis Bello López

**Affiliations:** 1 Health Research Institute of Santiago de Compostela (IDIS), Santiago de Compostela, Spain; 2 Division of Hematology, SERGAS, Complexo Hospitalario Universitario de Santiago de Compostela (CHUS), Santiago de Compostela, Spain; 3 University of Santiago de Compostela, Santiago de Compostela, Spain

**Keywords:** Cancer genomics, Chronic lymphocytic leukaemia

Dear Editor,

The analysis of hundreds of chronic lymphocytic leukemia (CLL) exomes has shed new light on the heterogeneous genomic background characterizing this disease^1,2^. At the same time, the increased availability of exome-sequencing data comes along with a big bottleneck in the interpretation of its results, which is related to the remarkable heterogeneity in mutation detection between different bioinformatic protocols. Differences in clonality, purity, sequencing coverage, and quality constitue difficulties for most variant callers. The methods with the highest sensitivity are frequently accompanied by lower precision, leading to remarkable differences in mutation detection^3–5^. Therefore, we hypothesize that numerous variants in large sequencing projects have passed unnoticed.

Here, we report the results of a complementary analysis performed on the *International Cancer Genome Consortium* (ICGC) CLL cohort^6^. The final analysis included 49 monoclonal B cell lymphocytosis and 390 treatment-naive CLL samples. Mutation detection was performed with two different methods: *VarsCan2*, which uses a heuristic/statistical method for variant detection; and *Platypus*, which implements a Bayesian approach and local realignment of reads for indel and complex mutation detection. Variant quality was recalibrated using a logistic model, and drivers were detected by integrating the results of methods based on mutation frequency (*MuSiC2*), functional impact (*OncodriveFM*), co-localization (*OncodriveClust* and *Mutation3D*), and pathogenicity prediction (*VEST* and *CHASM*) (Supplementary Methods). Cox regression was used for survival analysis. Assumption of proportional hazards was checked with Schoenfeld’s method. An unadjusted model was used to test the association of each mutated gene/pathway with time to treatment and overall survival. Similarly, we created an adjusted model which included variables associated with outcomes of interest at a nomial *p*-value < 0.2 (*IGHV* status, sex, and stage at diagnosis for time to treatment analysis; and *IGHV* status, age and stage at diagnosis for overall survival analysis). In the case of pathways analysis, the total number of mutations in genes belonging to each pathway were used as input. *P*-values were adjusted for multiple testing using the Benjamini–Hochberg (BH) method.

A total of 28,350 mutations were detected in 439 treatment-naive patient samples, of which 12,057 affected protein-coding regions (Supplementary Table 1). There were 8,965 non-silent and 3,095 silent mutations. The large majority of the non-silent mutations were missense (7,558 events). Point mutations were the most frequent (21,180), followed by short deletions (3,240) and insertions (2,041). There were 1,888 multi-nucleotide mutations (involving 2 or more consecutive nucleotides) (Supplementary Fig. 1).

Sixty-six genes were detected as putative drivers (Fig. 1, Supplementary Table 2, Supplementary Tables 3–8), of which thirty-two had been previously described by Puente et al.^1^ Among the novel ones, the most frequently mutated were *DTX1*, *LPHN3*, *LRP1B*, *LTB*, and *WDFY3*. *LPHN2* and *SI* were mutated in six patients; *BIRC6*, *DOCK1*, *MLL3*, *PCDH15*, *PTPN13*, *PTPRM*, *RELN*, and *TFEB* were mutated in five patients and the remaining putative drivers were mutated in four different cases. Furthermore, *WDFY3* harbored two additional silent mutations that are predicted to create new donor or acceptor cryptic sites. *BIRC6*, *DOCK1*, *KMT2C*/*MLL3*, *PTPRB*, and *PTPRT* were each affected by one silent mutation predicted to create a new cryptic splice site. Mutations in *IGLL5* were frequent and located in hotspots, but they were accompanied by a high rate of silent mutations. Finally, we observed that *FREM1* was targeted by four likely functional non-synonymous mutations and two additional silent mutations in the same position. Most of the new proposed drivers play well-defined roles in carcinogenesis, such as *EPHA7*;^7^ *MYCBP2*;^8^ *PTPRM*^9^. Other putative drivers have been linked to oncogenesis before, such as the autophagy regulator *WDFY3*^10^, the Notch pathway gene *DTX1*^11^, the latrophilin genes *LPHN2* and *LPHN3*^12^, as well as *FREM1*, which encodes the MYD88 and NFkB pathways related-protein TILRR^13^. Similarly, driver mutations in *CARD11* and *SI* have been previously described in CLL^2,14^, and the genes *BIRC6* and *KMT2C/MLL3* are paralogs of the CLL drivers *BIRC3* and *KMT2D*.

**Fig. 1:**
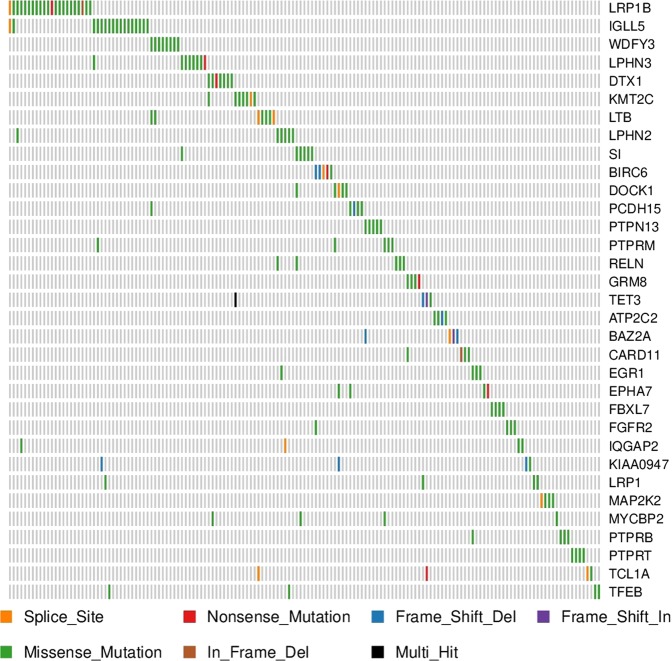
Mutation plot representing the most frequent novel CLL drivers identified in this study. Plot indicating the number and type of mutations in driver genes. Only drivers not described by Puente et al^1^. are represented

Low-frequency and likely pathogenic mutations in 60 genes (Supplementary Table 9) were detected. This type of mutations affected known cancer drivers (*EGFR*, *ERBB4*, *MAP2K1*, *NF1*, *NFKB1*, *NOTCH3*, and *SRSF1*), including multiple drivers of lymphoproliferation such as *BAX*, *BCOR*, *BCR*, *BTG2*, *DIS3*, *IKZF3*, *KRAS*, *PPM1D*, *PTPN11*, *SETD1B*, *TLR2*, and *TRAF3*. The list also includes regulators of lymphocyte pathways (*CD19*, *CD36*, *ALCAM*) and of relevant cancer pathways such as the Notch pathway (*NOTCH3*, *DMXL2*, and *SBNO1*), WNT/β-catenin pathway (*DACT1*); DNA polymerization (*POLE*) and epigenetic regulation (*KDM5A, HIST1H1D*, *PHF1* and single mutations at *HIST1H2BC* and *HIST1H2BG*). Moreover, isolated missense mutations in relevant oncogenes and tumor suppressor genes such as *EP300*, *KIT*, *MELK*, and *PTEN* were among the most significant events.

Non-synonymous mutations in 16 genes were significantly associated with time to first treatment (*q*-value < 0.1, Supplementary Table 10). The list included known CLL drivers such as *ATM*, *SF3B1*, *BRAF*, *NOTCH1*, *BIRC3, IRF4, and ZMYM3*, as well as other putative novel drivers such as *EPHA7* and *SI*. Mutations in *IGLV3-21*, *DOCK1*, and *EPHA7* were associated with time to treatment after covariate adjustment (*q*-value < 0.1, Supplementary Table 11). In order to assess the potential effect of silent mutations on time to treatment, we included them in the regression, revealing new significant associations in *IGHV1-69*, *IGKJ5*, *IGHV2-70*, and *FAT1*. Furthermore, silent mutations in *IGLV3-21* reduced the association *p*-value further (Supplementary Figure 2).Only two *IGLV3-21* mutated cases co-expressed *IGHV3-21*, indicating an independent role of the IGHV3-21/IGLV3-21 stereotyped B cell receptor. This is in concordance with a recent report about the adverse prognosis of *IGLV3-21* expression in CLL^15^. Finally, mutations in *ASXL1*, *ATM*, *IGHV1-69, SPEN*, *SF3F1, PLCH1, and POT1* were associated with overall survival (*q*-value < 10%, Supplementary Table 12), but none of these was significant after covariate adjustment (*q*-value < 0.1; Supplementary Table 13).

The genes *IGLL5*, *LTB*, *ZFP36L1*, *LRP1B*, and *PCDH15* were significantly enriched in intronic mutations (*q*-value < 0.1; Supplementary Table 14, Supplementary Table 15). Mutations in *ZFP36L1* and *DAPK1* were independently associated with time to first treatment (adjusted *q*-value < 0.1), whereas those in *IGHV3-49* were independently associated with overall survival (adjusted *q*-value < 0.1; Supplementary Tables 16–19).

A pathway-level inquiry detected 62 terms enriched in mutations (Bonferroni *p*-value < 0.1) (Supplementary Table 20). The most significantly mutated pathways were *“*RB pathway”, “TP53 pathway”, “ATM pathway”, “Apoptotic Signaling in Response to DNA Damage”, “TP53 Hypoxia pathway” and the “G1 pathway*”*. Most of the significant associations with clinical evolution were influenced by the presence of frequent driver mutations within the pathway. However, the following four significant pathways did not include any high-frequency CLL-driver gene: *“*CDK5 pathway”, “Apoptosis-induced DNA fragmentation”, “FRS2 mediated cascade”, and the “RAF MAP Kinase cascade*”*. We detected an interesting pattern in the *TP53 downstream pathway*, which affected ~10% of the patients. Mutations in this pathway were strongly and independently associated with shorter time to first treatment (*p*-value 3.80 × 10^−5^, Fig. 2a, b, Supplementary Table 21), and removing *TP53* mutated cases from the analysis did not affect the association substantially (*p*-value 5.3 × 10^−4^). These mutations were also significantly associated with lower overall survival (*p*-value 2.81 × 10^−4^), but not independently of *IGHV* status (*p*-value 0.54). These results suggest that the disruption of the *TP53* pathway plays an active role in CLL.

**Fig. 2:**
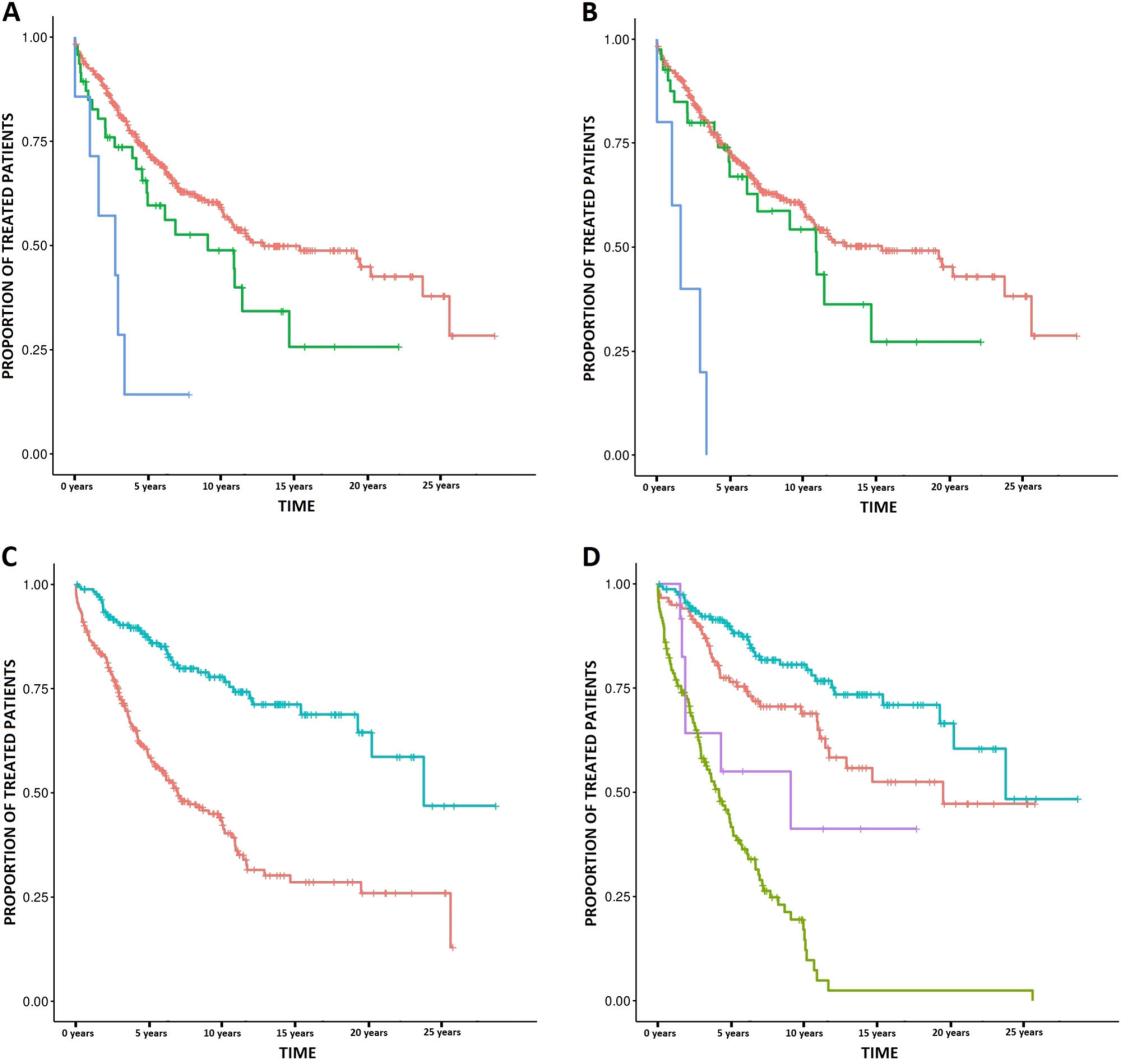
New prognostic biomarkers of CLL clinical evolution. **a–b** Kaplan–Meier plots indicating the association between mutations in the TP53 pathway and time to treatment with and without TP53 mutated cases (Fig. 2a, b, respectively). The red line indicates patients without mutation in this pathway, whereas the green and blue lines indicate patients with one or more than one mutation, respectively. **c–d** Kaplan–Meier plots indicating the association of mutations in the 3′ UTR and flanking region of *IGKC* with time to treatment. In Fig. 2c the red line indicates those patients without mutations in this region and the blue line indicate mutated cases. Similarly, in Fig. 2d the blue line indicates *IGHV* mutated cases with mutation in *IGKC*, the red line indicates *IGHV* mutated cases without *IGKC* mutation, the purple line indicates *IGHV* unmutated cases with *IGKC* mutation and the green line indicates those patients with both unmutated *IGHV* and *IGKC*

Finally, some analyzed non-coding regions located near immunoglobulin-related genes exhibited a remarkable mutation frequency. Mutations in the 3′ UTR of *IGHV1-69* were independently associated with lower time to treatment (adjusted *q*-value < 0.1, 95% HR 1.09–4.33). Furthermore, hypermutation events occurred in a 1,543 base pair region located in the 5′ flank and UTR region of *IGKC* (172 patients, 40% of the total population, Supplementary Tables 22–23). These mutations were strongly associated with longer time to first treatment (*p*-value 7.23 × 10^−11^, HR 0.21–0.44; Fig. 2c) and were independent of *IGHV* status, sex, and clinical stage at diagnosis (*p*-value 6.3 × 10^−3^, *q*-value 3.7 × 10^−2^, HR 0.39–0.86; Fig. 2d). Similarly, an association with longer overall survival was detected (*p*-value 2.81 × 10^−4^), but not independently of other covariates (*p*-value 0.54). This region includes protein-coding sequences of some immunoglobulin genes (namely *IGKJ1*, *IGKJ2*, *IGKJ3*, *IGKJ4*, and *IGJK5*). Although these genes were mutated in 126 cases, most of them (96%) had concurrent mutations in the surrounding non-coding region.

For mutation validation, we matched whole genome sequencing data available in a subset of 88 samples which was used. We could validate 94.38, 100, and 97.75% mutations located in new putative exonic and intronic drivers, as well as in 5′UTR region of *IGKC*, respectively (Supplementary Table 24). Importantly, all non-confirmed mutations were subclonal.

Some of our results need further clarification in future approaches. Particularly, the frequency, functional and clinical implications of the new putative drivers needs to be replicated in independent cohorts. Nevertheless, the novelty and relevance of some of our results anticipate important implications in the biological comprehension and prognostic stratification of CLL.

## Supplementary information

Supplementary Tables

Supplementary Table & Figure Legends

Supplementary Figure 1

Supplementary Figure 2

Supplementary Methods
