# Supplementary material for: Identification of new putative driver mutations and predictors of disease evolution in chronic lymphocytic leukemia
Source: Blood Cancer J. 2019 Sep 30;9(10):78. doi: 10.1038/s41408-019-0243-3 (PMC6769000; doi:10.1038/s41408-019-0243-3)
Supplement: Supplementary file 2 — Supplementary Table & Figure Legends [file 41408_2019_243_MOESM2_ESM.pdf]

**Supplementary Figure 1.** Upper left plot: results by mutation type. DNP= dinucleoitde mutation; TNP= trinucleotide mutation; SNP= single nucleotide mutation; ONP= oligo-nucleotide mutation; INS= insertion; DEL= deletion; MUL= multihit. Upper middle plot: representation of mutation frequencies by type. Upper right plot: frequency of point mutation types. Lower left and right plots: per-sample mutation distribution.

**Supplementary Figure 2.** Kaplan-Meier plot reflecting the association of *IGLV3-21* mutations with time to treatment. The blue line reflects mutated cases.

**Supplementary Methods.** Detailed information on the pipeline used for variant detection, filtering and driver detection can be consulted in this file.

**Supplementary Table 1.** Mutation data.

**Supplementary Table 2.** CLL drivers detected in this study. The detection method is indicated in the columns. Previously identified drivers by *Puente et al.* (2015) are labeled red, those identified by *Landau et al.* (2015) are labeled green and those reported by both studies are labeled blue. Clonal (VAF > 40%) and subclonal (VAF < 40%) mutations are indicated in the last column.

**Supplementary Table 3.** MuSiC2 analysis results of non-synonymous exonic mutations.

**Supplementary Table 4.** OncodriveFM analysis results of non-synonymous exonic mutations.

**Supplementary Table 5.** OncodriveClust analysis results of non-synonymous exon mutations.

**Supplementary Table 6.** mutation3D analysis results of non-synonymous exon mutations..

**Supplementary Table 7.** CHASM analysis results of non-synonymous exon mutations.

**Supplementary Table 8.** VEST analysis results of non-synonymous exon mutations.

**Supplementary Table 9.** Mutations at low frequency drivers, along with p-values for their predicted effect on protein function calculated by CHASM and VEST. Only mutations with q-values <0.25 are shown. Previously identified drivers by *Puente et al.* (2015) are labeled red,

those identified by *Landau et al.* (2015) are labeled green and those reported by both studies are labeled blue.

**Supplementary Tables 10-11.** Non-silent mutations association with treatment-free survival since diagnosis, unadjusted model and adjusted models respectively.

**Supplementary Table 12-13.** Non-silent mutations association with overall survival, unadjusted and adjusted models respectively.

**Supplementary Table 14.** Genes enriched in intronic mutations at a significant or suggestive level (MuSiC2 q-value <0.1 or <0.25, respectively).

**Supplementary Table 15.** MuSiC2 analysis results of intronic mutations.

**Supplementary Table 16-17.** Intronic mutations association with treatment-free survival since diagnosis, unadjusted and adjusted models respectively.

**Supplementary Tables 18-19.** Intronic mutations association with overall survival, unadjusted and adjusted models respectively.

**Supplementary Table 20.** PathScore analysis results of non-synonymous exon mutations.

**Supplementary Table 21.** Pathways level association with time to treatment and overall survival, unadjusted and adjusted models as stated in the headers.

**Supplementary Table 22.** 5’ UTRs+Flanks association with time to treatment and overall survival. Data for unadjusted and adjusted models are indicated according to the corresponding headers.

**Supplementary Table 23.** 3’ UTRs+Flanks association with time to treatment and overall survival. Data for unadjusted and adjusted models are indicated according to the corresponding headers.

**Supplementary Table 24.** Number of mutations included for validation and confirmed in the WGS data. Results for non-coding drivers, intronic drivers and the 5'UTR of *IGKC* are reported separately.
