# Supplementary figures and images for: Identification of new putative driver mutations and predictors of disease evolution in chronic lymphocytic leukemia
Source: Blood Cancer J. 2019 Sep 30;9(10):78. doi: 10.1038/s41408-019-0243-3 (PMC6769000; doi:10.1038/s41408-019-0243-3)

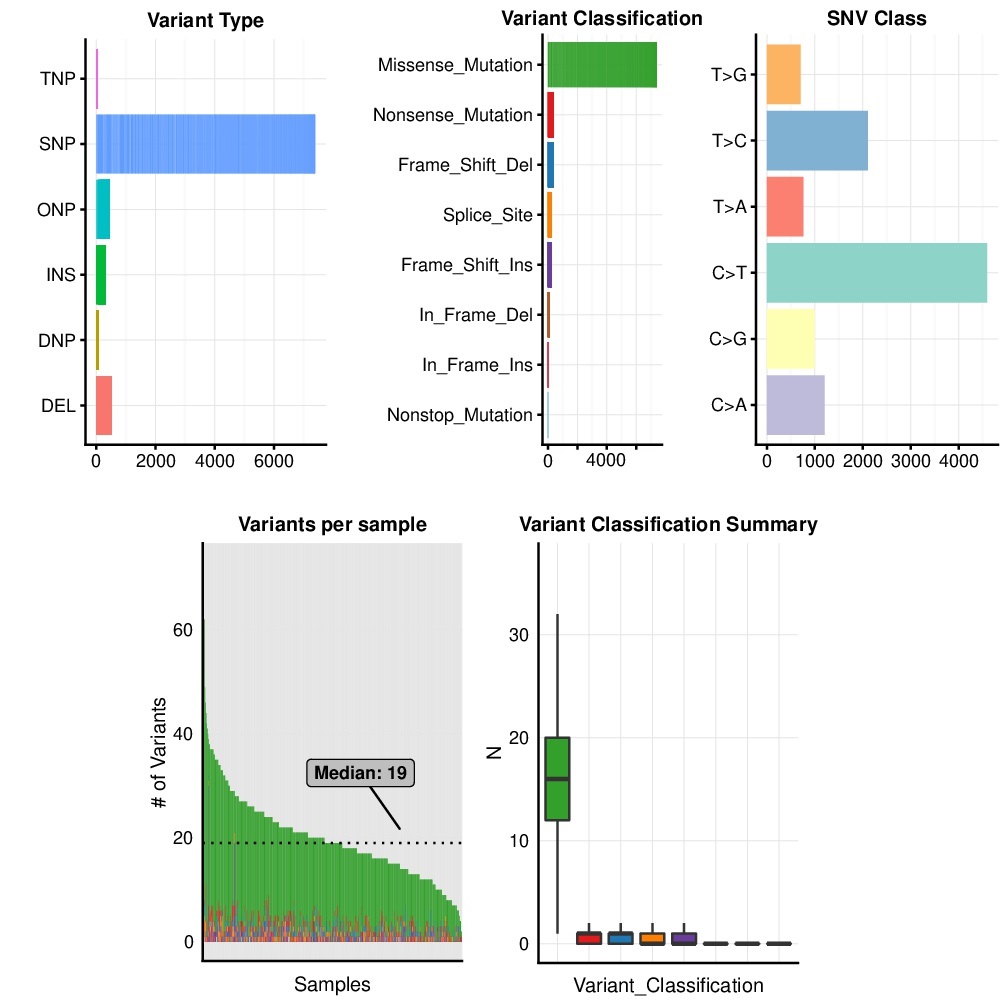

Supplement: Supplementary file 3 — Supplementary Figure 1 [file 41408_2019_243_MOESM3_ESM.jpg]

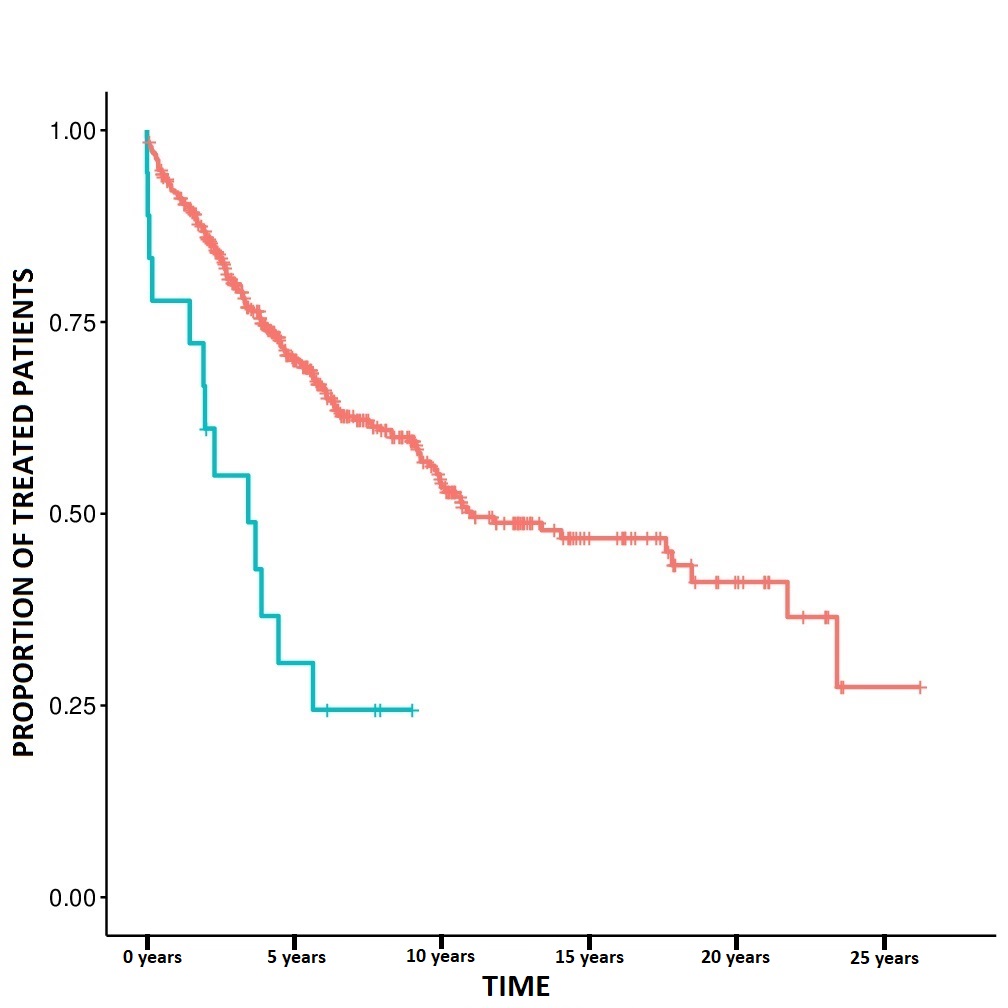

Supplement: Supplementary file 4 — Supplementary Figure 2 [file 41408_2019_243_MOESM4_ESM.jpg]
