# Supplementary material for: Identification of new putative driver mutations and predictors of disease evolution in chronic lymphocytic leukemia
Source: Blood Cancer J. 2019 Sep 30;9(10):78. doi: 10.1038/s41408-019-0243-3 (PMC6769000; doi:10.1038/s41408-019-0243-3)
Supplement: Supplementary file 5 — Supplementary Methods [file 41408_2019_243_MOESM5_ESM.pdf]

Supplementary Methods

1. Data preprocessing, variant detection, filtering and annotation

Exome-seq was performed by *Puente et al.* (2015) as described in their original paper. Briefly, 3 µg of genomic DNA were used for paired-end sequencing library construction, followed by enrichment in exomic sequences using the *SureSelect Human All Exon 50Mb* kit (Agilent Technologies). Next, DNA was pulled down using magnetic beads with streptavidin, followed by 18 cycles of amplification. Sequencing was performed on an Illumina GAIIx or on a HiSeq2000 sequencer (2x76bp). Reads were previously aligned to the reference genome (GRCh37.75) using *bwa* [1]. We performed duplicate read removal, sorting and indexing using *samtools* [2]. Base quality score recalibration was made with *BamUtil* [3] using a logistic regression model.

Samtools parameters were the following: "*C=50*"; "*d=250*", "*Q=13*", "*R*", "*O*", "*e=10*", "*F=0.002*", "*h=100*", "*m=1*", "*o=20*". The remaining parameters were run as in default mode. Next, VarScan2 [4] was run on paired-end mode with default parameters and the "*strand-filter*" option. For somatic variant detection, variants were filtered according to the following specifications: Fisher's p-value for variant frequency distribution between tumor and normal samples below 0.05, minimum coverage of 10x in tumor and control samples and a minimum mutation VAF of 10%. We discarded those mutations with a VAF in the control above 5%, more than 5 absolute reads covering the variant in the control and less than 5 variant reads in the tumoral sample (note that a small contamination of the control sample by CLL leukocytes is expected).

Platypus2 [5] was run with the following specifications: "*minVarFreq=0.02*", "*minReads=2*", "*maxReads=8000*", "*assemble=1*", "*minBaseQual=20*", "*trimSoftClipped=1*", "*minPosterior=20*", "*sbThreshold=0.01*", "*badReadsWindow=15*" and "*badReadsThreshold=15*". Variants labeled by platypus as "*HapScore*", "*SC*", "*strandBias*" and "*MQ*" were discarded. A minimum of 10 reads covering a position and 2 reads covering a variant were set for calling, a minimum genotype quality (GQ) of 20 Phred, genotype likelihood (GL) below -3, maximum homopolymer run (HP) below 11, minimum variant quality adjusted per read depth (QD) above 2 and minimum median minimum base quality for vases around variant (MMLQ) above 10. Somatic variants were selected according to the following filters: Fisher p-value for variant frequency distribution between tumor and normal samples below 0.05, a minimum variant allele frequency (VAF) of 10% and at least 5 reads covering the variant. We discarded those mutations with a VAF in the control above 5%, more than 5 absolute reads covering the variant in the control and less than 5 variant reads in the tumoral sample.

Variants from the two different callers were normalized and fused into single vcf files using *CombineVariants* functions implemented in the *Genome Analysis Toolkit* (GATK) [6]. Mutations were annotated using the *Variant Effect Predictor* (VEP) [7] and converted to MAF format [8]. Each mutation was annotated to *dbSNP* and *ExAC* databases. Mutation plots were produced using *maftools* [9].

2. Driver detection tools

2.1 MuSiC2 Analysis

We ran Music2 [10] analysis on coding and non-coding regions covered by *Agilent Exome SureSelect All Exon v4* kits with at least x10 depth in tumor and control samples. The GC-adjusted Convolution Test test was used as a measure of significance. Genes mutated in at least 4 patients with a Benjamini-Hochberg (BH)-adjusted p-value <0.1 were selected as potential new drivers. A similar procedure was used to analyzed intron mutation enrichment. Exome-seq also covers intronic regions in the neighbourhood of the exons, and mutations at these levels may be functional. To analyze gene enrichment in intronic mutations, we created background mutation statistics including all intronic regions that were covered with at a 10x depth in both tumor and normal samples.

2.2 OncodriveFM Analysis

We ran OncodriveFM [11] analysis with default parameters. Putative drivers were considered if they were mutated in at least 4 patients and had BH-adjusted p-values <0.1.

2.3 OncodriveClust

OncodriveClust [12] analysis for detection of mutation "hotspots" was run as implemented in *maftools* with default parameters. We set a mutation threshold of 4 events and a BH-adjusted p-value <0.1 in order to consider new drivers.

2.4 mutation3D analysis

Missense mutations were analyzed using mutation3D [13] to find genes whose missense mutations co-occur on specific tridimensional domains of the protein. The algorithm was run with the following parameters: minimum missense mutation number of 3, minimum number of mutations per cluster of 2, maximum intracluster distance of 20Å and MPQS >1.1. A BH-adjusted p-value threshold of 0.1 was selected as significance threshold.

2.5 CRAVAT analysis

We analyzed non-synonymous mutations with the CRAVAT pipeline [14], which included both the *Cancer-Specific High-throughput Annotation of Somatic Mutations* (CHASM) [15] and the *Variant Effect Scoring Tool* (VEST) [16] methods. Both of them are based on machine-learning (ML) classification of tumorigenic mutations based on a known set of driver mutations. Significant genes were selected as those with a BH-adjusted composite p-value <0.05.

Silent mutations at putative driver genes were analyzed with *Human Splicing Finder* [17] in search for donor or acceptor cryptic splice sites. Putative drives affecting less than 4 patients (circa 1% of the whole sample) and with more than 1 splice-neutral silent mutation were not considered for this analysis unless otherwise specified in the text. Genes were assessed for expression in lymphoid tissues using the *Human Protein Atlas* [18], and those without expression were discarded, except in the case of known human cancer drivers.

3. Low frequency putative drivers

CHASM and VEST methods were used to prioritize candidate driver mutations according to their predicted functional impact. Low frequency mutated genes present in less than 4 patients and with at least 2 linkely functional mutations (BH-adjusted p-value <0.25) were considered likely to be drivers if: 1) they represented >50% of the detected mutations in that gene; 2) the gene is expressed in lymphoid tissues and 3) had no synonymous mutations in the same gene, except for the case of *BCR* and *PTPN11*, which are known CLL drivers.

4. Pathways Analysis

PathScore analysis [19] was run with default mutation background frequency. To limit potential false findings due to false mutations we discarded all those that had a MAF above 0.01 in the *Non-Finish European Population* of ExAc. Significantly mutated pathways were labeled as those with Bonferroni adjusted p-value <0.1.

5. Mutation visual inspection and validation

Mutations were manually visualized using the Integrative Genomics Viewer [20]. For validation purposes, we used a subset of 88 whole genome sequencing (WGS) data that had matched exome sequencing data. We visually analyzed the subset of mutations falling at candidate driver genes (those that obtained BH q-values <0.25 in driver detection method, including intronic mutations, as well as those mutations in the list of new putative low-frequency drivers).

7. Code availability

The software used for this analysis is available in public repositories. Interested readers can contact the first author of this manuscript in order to obtain any further information.

1. Li H, Durbin R. Fast and accurate short read alignment with Burrows-Wheeler transform. Bioinformatics. 2009 Jul 15;25(14):1754-60. doi: 10.1093/bioinformatics/btp324. Epub 2009 May 18.
2. Li H, Handsaker B, Wysoker A, Fennell T, Ruan J, Homer N, Marth G, Abecasis G, Durbin R, and 1000 Genome Project Data Processing Subgroup, The Sequence alignment/map (SAM) format and SAMtools, Bioinformatics (2009) 25(16) 2078-9 [19505943]
3. Breese MR, Liu Y. NGSUtils: a software suite for analyzing and manipulating next-generation sequencing datasets. Bioinformatics. 2013;29(4):494–6. Epub 2013/01/15. pmid:23314324; PubMed Central PMCID: PMC3570212.
4. Koboldt DC, Zhang Q, Larson DE, Shen D, McLellan MD, Lin L, Miller CA, Mardis ER, Ding L, Wilson RK. VarScan 2: somatic mutation and copy number alteration discovery in cancer by exome sequencing. Genome Res. 2012 Mar;22(3):568-76. doi: 4.1101/gr.129684.111. Epub 2012 Feb 2.
5. Rimmer A, Phan H, Mathieson I, Iqbal Z, Twigg SRF; WGS500 Consortium, Wilkie AOM, McVean G, Lunter G. Integrating mapping-, assembly- and haplotype-based approaches for calling variants in clinical sequencing applications. Nat Genet. 2014 Aug;46(8):912-918. doi: 10.1038/ng.3036. Epub 2014 Jul 13.
6. Van der Auwera GA, Carneiro MO, Hartl C, Poplin R, Del Angel G, Levy-Moonshine A, Jordan T, Shakir K, Roazen D, Thibault J, Banks E, Garimella KV, Altshuler D, Gabriel S, DePristo MA. From FastQ data to high confidence variant calls: the Genome Analysis Toolkit best practices pipeline. Curr Protoc Bioinformatics. 2013;43:11.10.1-33. doi: 10.1002/0471250953.bi1110s43.

7. McLaren W, Gil L, Hunt SE, Riat HS, Ritchie GR, Thormann A, Flicek P, Cunningham F. The Ensembl Variant Effect Predictor. Genome Biology Jun 6;17(1):122. (2016) doi:10.1186/s13059-016-0974-4
8. Kandoth C. Convert a VCF into a MAF, where each variant is annotated to only one of all possible gene isoforms, (2018), GitHub repository, https://github.com/mskcc/vcf2maf
9. Mayakonda, A, Koeffler, HP. Maftools: Efficient analysis, visualization and summarization of MAF files from large-scale cohort based cancer studies. bioRxiv (2016). doi: http://dx.doi.org/10.1101/052662
10. Dees ND, Zhang Q, Kandoth C, Wendl MC, Schierding W, Koboldt DC, Mooney TB, Callaway MB, Dooling D, Mardis ER, Wilson RK, Ding L. MuSiC: identifying mutational significance in cancer genomes. Genome Res. 2012 Aug;22(8):1589-98. doi: 10.1101/gr.134635.111. Epub 2012 Jul 3.
11. Gonzalez-Perez A, Perez-Llamas C, Deu-Pons J, Tamborero D, Schroeder MP, Jene-Sanz A, Santos A, Lopez-Bigas N. IntOGen-mutations identifies cancer drivers across tumor types. Nat Methods. 2013 Nov;10(11):1081-2. doi: 10.1038/nmeth.2642. Epub 2013 Sep 15.
12. Tamborero D, Gonzalez-Perez A, Lopez-Bigas N. OncodriveCLUST: exploiting the positional clustering of somatic mutations to identify cancer genes. Bioinformatics. 2013 Sep 15;29(18):2238-44. doi: 10.1093/bioinformatics/btt395. Epub 2013 Jul 24.
13. Meyer MJ, Lapcevic R, Romero AE, Yoon M, Das J, Beltrán JF, Mort M, Stenson PD, Cooper DN, Paccanaro A, Yu H. mutation3D: Cancer Gene Prediction Through Atomic Clustering of Coding Variants in the Structural Proteome. Hum Mutat. 2016 May;37(5):447-56. doi: 10.1002/humu.22963. Epub 2016 Feb 18.
14. Masica DL, Douville C, Tokheim C, Bhattacharya R, Kim R, Moad K, Ryan MC, Karchin R. CRAVAT 4: Cancer-Related Analysis of Variants Toolkit. Cancer Res. 2017 Nov 1;77(21):e35-e38. doi: 10.1158/0008-5472.CAN-17-0338.
15. Wong WC, Kim D, Carter H, Diekhans M, Ryan MC, Karchin R. CHASM and SNVBox: toolkit for detecting biologically important single nucleotide mutations in cancer. Bioinformatics. 2011 Aug 1;27(15):2147-8. doi: 10.1093/bioinformatics/btr357. Epub 2011 Jun 17.
16. Douville C, Masica DL, Stenson PD, Cooper DN, Gygax DM, Kim R, Ryan M, Karchin R. Assessing the Pathogenicity of Insertion and Deletion Variants with the Variant Effect Scoring Tool (VEST-Indel). Send to Hum Mutat. 2016 Jan;37(1):28-35. doi: 10.1002/humu.22911. Epub 2015 Oct 26.
17. Desmet FO, Hamroun D, Lalande M, Collod-Béroud G, Claustres M, Béroud C. Human Splicing Finder: an online bioinformatics tool to predict splicing signals. Nucleic Acids Res. 2009 May;37(9):e67. doi: 10.1093/nar/gkp215. Epub 2009 Apr 1.
18. Thul PJ, Åkesson L, Wiking M, Mahdessian D, Geladaki A, Ait Blal H, Alm T, Asplund A, Björk L, Breckels LM, Bäckström A, Danielsson F, Fagerberg L, Fall J, Gatto L, Gnann C, Hober S, Hjelmare M, Johansson F, Lee S, Lindskog C, Mulder J, Mulvey CM, Nilsson P, Oksvold P, Rockberg J, Schutten R, Schwenk JM, Sivertsson Å, Sjöstedt E, Skogs M, Stadler C, Sullivan DP, Tegel H, Winsnes C, Zhang C, Zwahlen M, Mardinoglu A, Pontén F, von Feilitzen K, Lilley KS, Uhlén M, Lundberg E. A subcellular map of the human proteome. Science. 2017 May 26;356(6340). pii: eaal3321. doi: 10.1126/science.aal3321. Epub 2017 May 11.
19. Gaffney SG, Townsend JP. PathScore: a web tool for identifying altered pathways in cancer data. Bioinformatics. 2016 Dec 1;32(23):3688-3690. Epub 2016 Aug 8.
20. Robinson JT, Thorvaldsdóttir H, Winckler W, Guttman M, Lander ES, Getz G, Mesirov JP. Integrative genomics viewer. Nat Biotechnol. 2011 Jan;29(1):24-6. doi: 10.1038/nbt.1754.
